# Effect of Antipyretic Therapy on Mortality in Critically Ill Patients with Sepsis Receiving Mechanical Ventilation Treatment

**DOI:** 10.1155/2017/3087505

**Published:** 2017-03-12

**Authors:** Sheng Ye, Dan Xu, Chenmei Zhang, Mengyao Li, Yanyi Zhang

**Affiliations:** ^1^Pediatric Intensive Care Unit, The Children's Hospital, Zhejiang University School of Medicine, Hangzhou 310003, China; ^2^Psychological Department, The Children's Hospital, Zhejiang University School of Medicine, Hangzhou 310003, China

## Abstract

*Purpose*. The study aimed to investigate the effectiveness of antipyretic therapy on mortality in critically ill patients with sepsis requiring mechanical ventilation.* Methods*. In this study, we employed the multiparameter intelligent monitoring in intensive care II (MIMIC-II) database (version 2.6). All patients meeting the criteria for sepsis and also receiving mechanical ventilation treatment were included for analysis, all of whom suffer from fever or hyperthermia. Logistic regression model and R language (R version 3.2.3 2015-12-10) were used to explore the association of antipyretic therapy and mortality risk in critically ill patients with sepsis receiving mechanical ventilation treatment.* Results*. A total of 8,711 patients with mechanical ventilator were included in our analysis, and 1523 patients died. We did not find any significant difference in the proportion of patients receiving antipyretic medication between survivors and nonsurvivors (7.9% versus 7.4%, *p* = 0.49). External cooling was associated with increased risk of death (13.5% versus 9.5%, *p* < 0.001). In our regression model, antipyretic therapy was positively associated with mortality risk (odds ratio [OR]: 1.41, 95% CI: 1.20–1.66, *p* < 0.001).* Conclusions*. The use of antipyretic therapy is associated with increased risk of mortality in septic ICU patients requiring mechanical ventilation. External cooling may even be deleterious.

## 1. Introduction 

Sepsis is a major threat to human health and is among the most important causes of morbidity and mortality in the intensive care unit (ICU). In 2010, sepsis accounted for approximately 5% of deaths in England [1]. Sepsis is defined as a life-threatening organ dysfunction caused by a dysregulated host response to infection. In the present time, organ dysfunction is defined in terms of a change in baseline Sequential Organ Failure Assessment (SOFA) score [2].

Fever, the cardinal symptom of sepsis, is common among patients admitted to the ICU. Some clinicians believe that fever is deleterious because it exacerbates the imbalance between oxygen supply and demand, and most will prescribe antipyretic therapy for fever control in order to relieve the symptom. In contrast, some clinicians consider fever as a protective response that can inhibit the growth of microorganisms, and suppression of fever may delay the recovery. These investigators do not recommend routine use of fever control in ICU patients with sepsis.

The management of fever induced by sepsis varies substantially across different institutions and hospitals [3, 4]. Circiumaru et al. conducted the first study on the relationship between fever and mortality in critically ill patients during their ICU stays and found that fever lasting more than 5 days was associated with increased mortality (*p* < 0.001) [5]. Barie et al. [6] were the first to study mortality risk in critically ill patients and recognized peak temperature as an important predictor of increased ICU mortality (*p* < 0.001). The most frequent ICU-acquired infection is ventilator-associated pneumonia [5–8], which can significantly increase patient length of stay, treatment costs, and mortality [9–12]. However, investigations into the effect of antipyretic therapy on mortality in patients with sepsis, especially those receiving mechanical ventilation treatment, are limited. One meta-analysis focused on the effect of antipyretic therapy on mortality in febrile ICU patients and reported no difference between patients treated with and those without antipyretic therapy [13]. The results of observational studies and randomized controlled trials (RCT) are conflicting due to variations in study population, design, and methods of antipyretic therapy [14]. We assume that the effect of antipyretic therapy on mortality would be influenced by age, SOFA score, or other variables. A large clinical database was utilized in our study.

## 2. Methods 

### 2.1. Patients

All patients meeting the criteria for sepsis and also receiving mechanical ventilation treatment were included for our study, all of whom suffer from fever or hyperthermia. Sepsis was defined according to the new consensus of the European Society of Intensive Care Medicine and the Society of Critical Care Medicine published in February 2016, with diagnosis based on the combination of infection and SOFA score ≥ 2 points [2]. The infection was defined if one of the following criteria was fulfilled: (1) ICD9 contains the term “infection” and “pneumonia,” “lung,” “abdomen,” “bloodstream,” or “renal or genitourinary tract” and (2) positive microbiological culture. All patients included in the present study had at least one recorded temperature greater than 37.2°C. Data management was performed by using the R language (R version 3.2.3 2015-12-10).

### 2.2. Study Protocol

The MIMIC-II (multiparameter intelligent monitoring in intensive care II) database (version 2.6) was employed for our study, which comprises deidentified health-related data associated with more than 40,000 patients who stayed in critical care units of the Beth Israel Deaconess Medical Center (Boston, MA) from 2001 to 2008. The MIMIC-II database includes demographics, vital signs, laboratory tests, medications, and other information. The author Z. Y. Y. obtained access to the database after completion of the NIH web-based training course “Protecting Human Research Participants.” Data extraction was performed by using structure query language (SQL) with Navicat Premium Version 10.0.7. When the data were extracted, we considered antipyretic therapy to consist of antipyretic medication and external cooling. The former included drugs such as ibuprofen, acetaminophen, naproxen, ketoprofen, voltaren, diclofenac, and nimesulide. The latter included cooling blankets and ice packs. The SQL to extract body temperature measurements was as follows.

SELECT value1num, charttime, icustay_id FROM chartevents WHERE itemid = 677. From this query we obtained 783,632 body temperature measurements, representing all the measurements of body temperature recorded in the database at various sites of the body. ICU mortality, a solid outcome, was the main outcome criterion. Other variables including SOFA, age, Simplified Acute Physiology Score- (SAPS-) 1 [15], lactate levels, care unit type, and sex were also extracted.

### 2.3. Outcome Measures

The primary outcome measure was ICU mortality. Other variables such as SAPS-1 and SOFA scores, antipyretic therapy data, lactate levels, and care unit type were also included. The SAPS-1 is a disease severity classification system, which is valuable in that it can be averaged for a group of patients, and the calculation results in a predicted mortality. Its name stands for “Simplified Acute Physiology Score.” The SOFA score is used to track a patient's status during ICU admission. It is a system to determine the extent of a person's organ function [16, 17].

### 2.4. Statistical Analysis

Variables were expressed as mean and standard deviation (SD), counts and percentages, or median and interquartile range [18]. As an effective prognostic indicator and evaluator for patient progress in ICU, the SOFA score was included in the model as one of the most important variables. The initial SOFA score is strongly correlated with mortality. Therefore, in the present study we extracted the sofa_first data.

Logistic regression model was used to examine the effect of antipyretic therapy on mortality in critically ill patients with sepsis receiving mechanical ventilation treatment [19]. The odds ratio of antipyretic therapy was calculated, with an OR > 1 indicating a positive coefficient of the antipyretic therapy group compared to the nonantipyretic therapy group. All statistical analyses were performed by using R language (R version 3.2.3 2015-12-10). A *p* value < 0.05 was considered significant.

## 3. Results 

A total of 40,000 ICU patients were included in the MIMIC-II database (version 2.6), including 8,711 patients meeting the criteria of sepsis and also requiring mechanical ventilation.

In Table 1, many variables significantly differed between survivors and nonsurvivors. Survivors were younger than nonsurvivors. As expected, nonsurvivors had significantly higher SOFA and SAPS-1 scores. Admission to a MICU was associated with increased risk of death (50.3% versus 39%, *p* < 0.001), whereas patients in a CSRU were less likely to die (21.6% versus 35.4%, *p* < 0.001). External cooling was associated with increased risk of death (13.5% versus 9.5%, *p* < 0.001). However, there was no significant difference in the proportion of patients receiving antipyretic medication between survivors and nonsurvivors (7.9% versus 7.4%, *p* = 0.49).

As shown in Table 2, we conducted logistic regression analysis to adjust for confounding factors of antipyretic therapy by importing prespecified variables (age, SAPS-1, SOFA score, sex, care unit, and antipyretic therapy). In this regression model, antipyretic therapy was positively associated with mortality risk (odds ratio [OR]: 1.41, 95% CI: 1.20–1.66, *p* < 0.001) (OR = odds ratios).

**Figure 1:**
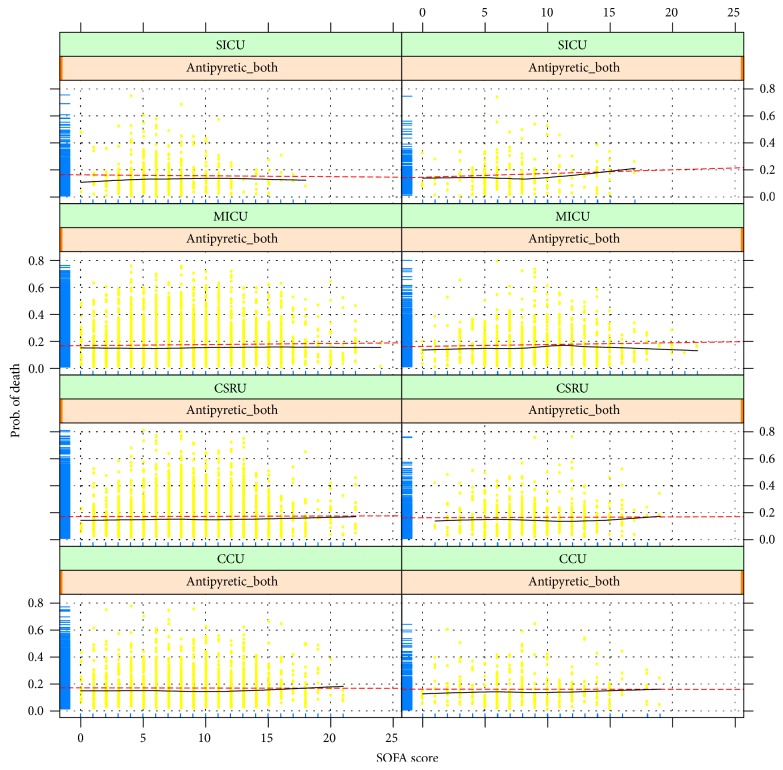


Figure 1 shows the relationship between SOFA score and probability of death across different ICU sectors, with or without antipyretic therapy (antipyretic_both means external cooling and drug cooling). Probability of death for the fitted model was plotted on the *y*-axis and SOFA score on the *x*-axis. Here the red dotted line, which was fitted by parametric method, represents the predicted fitted value, and the result is shown with a black curved line fitted by nonparametric method. As shown in the figure, there was no obvious change, but there was a statistically significant difference between SOFA score and probability of death. Therefore, we assume that the impact of antipyretic therapy on mortality for patients with different SOFA scores is quite small.

**Figure 2:**
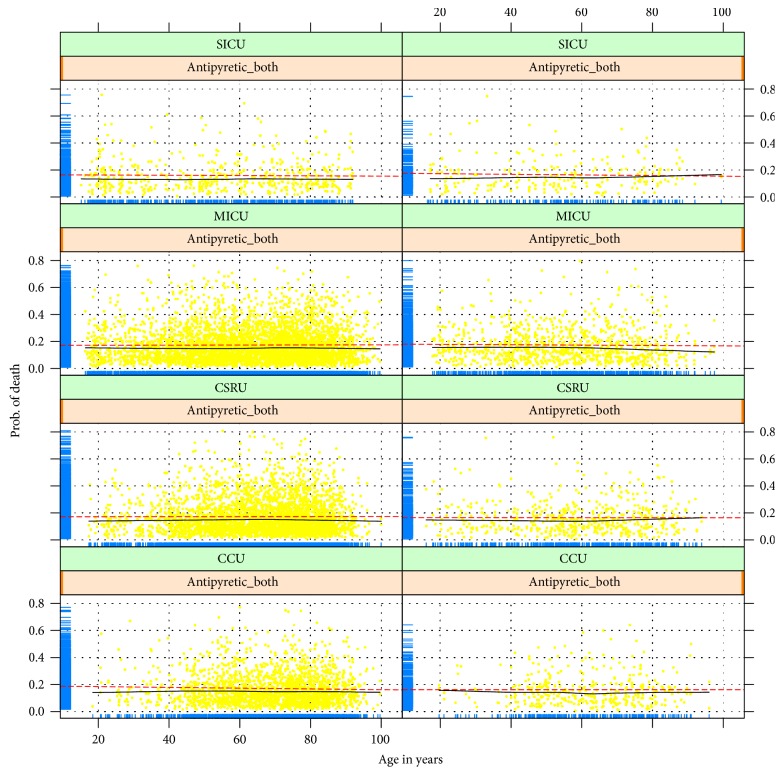


Figure 2 shows the plot of jittered outcome to reflect the relationship between age and probability of death. Probability of death was plotted on the *y*-axis for the fitted model and age on the *x*-axis. The classification of the model appears good in that most survivors have an estimated probability of death less than 0.2. As shown in the figure, there was no obvious change, but there was a statistically significant difference between age and probability of death. Therefore, we assume that the impact of antipyretic therapy on mortality for patients of different ages is quite small.

**Figure 3:**
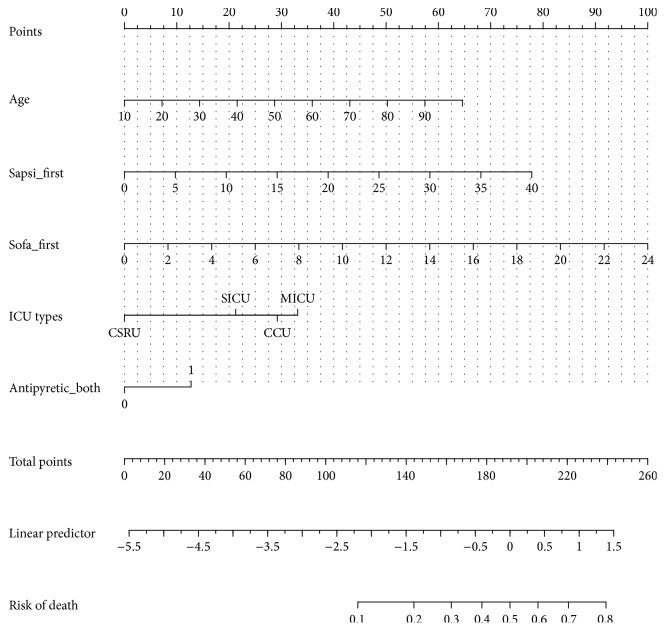
Figure nomogram.

Figure 3 shows the nomogram for prediction of the risk of death for ICU patients with sepsis. Each variable was represented by a bar. A given value of a variable can be mapped to the point bar at the top of the graph and there is a point value for that given value. After each variable is assigned a point number, they are summed and mapped to the total point bar. Then there will be a value in the “risk of death” bar corresponding to those total points. For instance, you have a septic patient aged 50 (point = 29), with SAPSI of 25 (point = 48), SOFA of 12 (point = 50), from MICU (point = 55), and treated with antipyretic therapy (point = 13). The total points approximate 29 + 48 + 50 + 55 + 13 = 195, which corresponds to 54% probability of death.

## 4. Discussion 

Evaluation of patient status before ICU admission is essential to ensure proper interventions and management of hospital resources. Variables including SOFA score, age, SAPS-1, lactate level, care unit type, and sex were crucial for the evaluation. Fever is common in septic ICU patients who receive mechanical ventilation treatment, so the protocol for fever control is vital to critically ill patients.

Our study indicated that antipyretic therapy had an adverse impact on mortality in critically ill patients with sepsis receiving mechanical ventilation treatment. We found that antipyretic therapy was positively associated with mortality risk (OR: 1.41, 95% CI: 1.20–1.66, *p* < 0.001; Table 2). This finding supports the hypothesis that hyperthermia is a natural response to infection and thus beneficial to septic patients who undergo mechanical ventilation treatment. It is plausible that the increased production of heat shock proteins, which are produced at the highest rate at high temperatures, could directly inhibit the growth of microorganisms and enhance immune function [20]. Some observational studies have shown that hyperthermia may confer protection against adverse outcome. In a study involving 612 patients with confirmed Gram-negative bacteria, fever within 24 hours was shown to be protective against mortality risk [21]. The FACE (Fever and Antipyretic in Critically Ill Patients Evaluation) investigators found that septic patients with a temperature above 39.5°C exhibited a downward trend in 28-day mortality [22]. A 2013 meta-analysis including 399 patients from five randomized trials found no survival benefit for antipyretic therapy in febrile critically ill patients (acute neurological injury excluded) [13], which is consistent with the results of our study. Randomized controlled trails are limited in sample size, which may lead to sampling error. Our study was based on data mining of critical care data, which can avoid the limitation of sample size.

As shown in Table 1, external cooling was associated with increased risk of death (13.5% versus 9.5%, *p* < 0.001), while there was no significant difference in the proportion of patients using antipyretic medications between survivors and nonsurvivors (7.9% versus 7.4%, *p* = 0.49). External cooling lowers the skin temperature and usually leads to muscle shivering, which can increase the metabolic rate, energy expenditure, and oxygen consumption. Therefore, external cooling tends to have greater adverse effects on mortality in critically ill patients with sepsis receiving mechanical ventilation compared to drug cooling, as shown in our study.

There were several limitations in the present study that should be acknowledged. The first one was that antipyretic therapy with drug and external cooling were combined in the multivariable analysis. In fact, the therapeutic effect of these two methods can be different. However, because some patients received both strategies for antipyretic treatment, it was difficult to disentangle the effects of these two treatments on mortality outcome. Further prospective trials may be conducted to investigate the specific effect of external cooling versus drug treatment.

Overall, our study found no beneficial effect of antipyretic therapy to reduce mortality risk in septic ICU patients requiring mechanical ventilation. External cooling may even be harmful. Since fever is very common in ventilated ICU patients with sepsis and antipyretic therapy may alter outcome, a large RCT comparing different fever control strategy in critically ill patients is urgently needed.

## 5. Conclusion

The use of antipyretic therapy is not beneficial for reducing mortality risk in septic ICU patients requiring mechanical ventilation. External cooling may even be deleterious.

## Figures and Tables

**Table 1:** Comparisons between survivors and nonsurvivors.

Variables	Overall (*n* = 8711)	Survivors (*n* = 7188)	Nonsurvivors (*n* = 1523)	*p*
Age (years)	66.1 (52.0, 77.5)	64.6 (50.9, 76.6)	71.3 (58.1, 81.2)	<0.001
SAPS-1	17 (14, 20)	16 (13, 20)	19 (16, 23)	<0.001
SOFA	8 (6, 11)	8 (6, 10)	10 (7, 14)	<0.001
Lactate level	0.84 ± 0.76	0.85 ± 0.79	0.75 ± 0.62	0.0002
Sex (male, %)	4866 (55.9)	4032 (56.1)	834 (54.8)	0.36
Care unit type^*∗*^ (%)				<0.001
MICU	3567 (40.9)	2801 (39.0)	766 (50.3)	
SICU	621 (7.1)	546 (7.6)	75 (4.9)	
CCU	1646 (18.9)	1293 (18.0)	353 (23.2)	
CSRU	2877 (33.0)	2548 (35.4)	329 (21.6)	
External cooling (*n*, %)	892 (10.2)	686 (9.5)	206 (13.5)	<0.001
Drug cooling (*n*, %)	652 (7.5)	531 (7.4)	121 (7.9)	0.49
Any antipyretic (*n*, %)	1385 (15.9)	1102 (15.3)	283 (18.6)	0.002

MICU, medical intensive care unit; SICU, surgical intensive care unit; CCU, coronary care unit; CSRU, cardiac surgery recovery unit; SAPS, Simplified Acute Physiology Score; SOFA, Sequential Organ Failure Assessment.

**Table 2:** Logistic regression model adjusting for confounding factors of antipyretic therapy.

Variables	OR	Lower limit	Upper limit	*p*
Age	1.01	1.01	1.02	<0.001
SAPS-1	1.07	1.05	1.08	<0.001
SOFA	1.13	1.11	1.15	<0.001
Female versus male	1.05	0.93	1.19	0.438
Care unit (versus MICU as reference)				
SICU	0.66	0.50	0.86	0.003
CCU	0.92	0.78	1.07	0.273
CSRU	0.39	0.33	0.45	<0.001
Antipyretic therapy (any)	1.41	1.20	1.66	<0.001

OR, odds ratio; SAPS-1, Simplified Acute Physiology Score-1; SOFA, Sequential Organ Failure Assessment; MICU, medical intensive care unit; SICU, surgical intensive care unit; CCU, coronary care unit; CSRU, cardiac surgery recovery unit.
